# Continuous Developmental and Early Life Trichloroethylene Exposure Promoted DNA Methylation Alterations in Polycomb Protein Binding Sites in Effector/Memory CD4^+^ T Cells

**DOI:** 10.3389/fimmu.2019.02016

**Published:** 2019-08-28

**Authors:** Stephanie D. Byrum, Charity L. Washam, John D. Patterson, Kanan K. Vyas, Kathleen M. Gilbert, Sarah J. Blossom

**Affiliations:** ^1^Department of Biochemistry and Molecular Biology, Arkansas Children's Research Institute, University of Arkansas for Medical Sciences, Little Rock, AR, United States; ^2^College of Medicine, University of Arkansas for Medical Sciences, Little Rock, AR, United States; ^3^Department of Pediatrics, Arkansas Children's Research Institute, University of Arkansas for Medical Sciences, Little Rock, AR, United States; ^4^Department of Microbiology and Immunology, Arkansas Children's Research Institute, University of Arkansas for Medical Sciences, Little Rock, AR, United States

**Keywords:** polycomb, trichloroethylene, CD4^+^ T cell, DNA methylation, developmental exposure

## Abstract

Trichloroethylene (TCE) is an industrial solvent and drinking water pollutant associated with CD4^+^ T cell-mediated autoimmunity. In our mouse model, discontinuation of TCE exposure during adulthood after developmental exposure did not prevent immunotoxicity. To determine whether persistent effects were linked to epigenetic changes we conducted whole genome reduced representation bisulfite sequencing (RRBS) to evaluate methylation of CpG sites in autosomal chromosomes in activated effector/memory CD4^+^ T cells. Female MRL+/+ mice were exposed to vehicle control or TCE in the drinking water from gestation until ~37 weeks of age [postnatal day (PND) 259]. In a subset of mice, TCE exposure was discontinued at ~22 weeks of age (PND 154). At PND 259, RRBS assessment revealed more global methylation changes in the continuous exposure group vs. the discontinuous exposure group. A majority of the differentially methylated CpG regions (DMRs) across promoters, islands, and regulatory elements were hypermethylated (~90%). However, continuous developmental TCE exposure altered the methylation of 274 CpG sites in promoters and CpG islands. In contrast, only 4 CpG island regions were differentially methylated (hypermethylated) in the discontinuous group. Interestingly, 2 of these 4 sites were also hypermethylated in the continuous exposure group, and both of these island regions are associated with lysine 27 on histone H3 (H3K27) involved in polycomb complex-dependent transcriptional repression via H3K27 tri-methylation. CpG sites were overlapped with the Open Regulatory Annotation database. Unlike the discontinuous group, continuous TCE treatment resulted in 129 DMRs including 12 unique transcription factors and regulatory elements; 80% of which were enriched for one or more polycomb group (PcG) protein binding regions (i.e., SUZ12, EZH2, JARID2, and MTF2). Pathway analysis of the DMRs indicated that TCE primarily altered the methylation of genes associated with regulation of cellular metabolism and cell signaling. The results demonstrated that continuous developmental exposure to TCE differentially methylated binding sites of PcG proteins in effector/memory CD4^+^ cells. There were minimal yet potentially biologically significant effects that occurred when exposure was discontinued. These results point toward a novel mechanism by which chronic developmental TCE exposure may alter terminally differentiated CD4^+^ T cell function in adulthood.

## Introduction

As many as 5–7% of Americans suffer from a group of disorders consisting of over 100 different diseases collectively called immune-mediated inflammatory diseases (IMIDs) that include hypersensitivity disorders and autoimmune diseases (1). These chronic, incurable disorders disproportionately affect females, and are among the leading causes of death among young and middle-age women (2). Although these diseases result in different types of tissue damage, they appear to share some common inflammatory pathways. In many cases this includes sustained T cell activation. While it is not known what causes IMIDs, the increased prevalence and incidence rates of autoimmune disease parallel the documented increase chemicals that pollute the environment. Thus, pollutants common to industrialized nations are increasingly being recognized as possible triggers for immunotoxicity and autoimmunity (3). One potential environmental risk factor is trichloroethylene (TCE) (4–7). TCE is an organic solvent best known for its use as an industrial chemical and metal degreaser. Because of improper disposal over the years, TCE has contaminated many water systems in the US. Based on likelihood of exposure and an increasing appreciation of its toxicity, TCE is on the list of the top 90 chemicals selected from ~85,000 in the Toxic Substances Control Act (TSCA) Inventory as having the highest potential for exposure and hazard (8).

The mechanisms behind TCE's ability to promote autoimmunity and hypersensitivity is not known. However, studies have shown that CD4^+^ T cells are especially sensitive to TCE's effects, and even if overt disease is not diagnosed, altered numbers of peripheral blood CD4^+^ T cells are often found in humans exposed to TCE (9–11). Expansion of peripheral blood CD4^+^ T cells is a biomarker for patients with occupational TCE hypersensitivity syndrome (12). As shown by ourselves and others, chronic adult TCE exposure in mice modulates the percentage of IFN-γ- and IL-17-secreting effector/memory CD4^+^ T cells in mice that went on to develop autoimmune hepatitis (13–15). Such effector/memory CD4^+^ T cell subsets have been shown to be important in promoting idiopathic and experimental autoimmune disease (16, 17).

Prevention of TCE-mediated autoimmune disease depends on a better understanding mechanisms responsible for disease initiation or progression. Effector/memory CD4^+^ T cells are the main drivers of autoimmune diseases due to their persistence and diverse contributions to pathology. In recent years it has been reported that the autoreactivity of effector/memory CD4^+^ T cells may be regulated at the level of DNA methylation. T cell methylation abnormalities are common in autoimmune disease, and widely documented in lupus patients and lupus mouse models. In lupus, disease progression was reportedly accompanied by global DNA hypomethylation, presumably by favoring expression of proinflammatory genes (18). However, both hypomethylated and hypermethylated CpGs have been documented in lupus T cells in several genome-wide DNA methylation studies (19, 20). Along these lines, administration of 5-azacytidine, a potent DNA methylation inhibitor, both promoted and suppressed autoimmunity in lupus prone mice (21, 22). These studies underscore the complexities associated with DNA methylation events associated with T cells in autoimmunity, and it is not surprising that both hypomethylation of inflammatory elements and hypermethylation of regulatory elements have been reported to occur in T cells during the course of disease (23, 24).

In our mouse model, we previously reported that TCE exposure altered DNA methylation in activated effector/memory cells, and this effect was not observed in naïve CD4s. In addition, unlike in naïve CD4s, both chronic and sub-chronic adult-only TCE exposure *in vivo* altered global and gene-specific DNA methylation in effector/memory CD4^+^ T cells using targeted bisulfite next-generation sequencing [(NGS) (25–27)]. More recently, genome-wide reduced representation bisulfite sequencing [RRBS) was used to interrogate activated effector/memory CD4^+^ T cells isolated from adult female MRL+/+ mice exposed to TCE for 40 weeks (28). A majority of the differentially methylated CpG regions (DMRs) significantly altered by TCE were regions associated with polycomb group (PcG) proteins. PcG-mediated epigenetic gene regulation requires the action of 2 different polycomb repressive protein complexes (PRCs): PRC1 and PRC2. PRC2 consists of core components JARID2 (Jumonji and AT-Rich Interaction Domain containing 2), SUZ12 (Suppressor of Zeste 12 protein Homolog), EED (Embryonic Ectoderm Development, and either Enhancer of Zeste Homolog (EZH) 2 or EZH1. The EZH paralogs have methyltransferase activity and are the only enzymes known to trimethylate histone H3 at lysine 27 *in vivo*. Trimethylated histone H3K27 (H3K27me3) is a PcG-specific chromatin modification that is widely present in the promoter regions of silenced genes and thought to provide PRC2 with a role in transcriptional repression (29). In T cells, PcG proteins are important in modulating regulatory T cell (T reg) function and effector cell differentiation and function (30, 31).

While our previous RRBS study revealed important new information for a potential role for PcG proteins in TCE-induced immunotoxicity, the experiment was conducted in mice exposed to TCE during adulthood. Because sensitivity to immunotoxicants is thought to be greater in animal models if exposure occurred during development compared to adulthood, we hypothesized that methylation changes in activated effector/memory CD4s would be more robust if exposure occurred during development and/or early life. Additionally, we have shown autoimmune pathology and a number of altered immunological effects were sustained in adult mice after developmental exposure after TCE was removed from the drinking water 15 weeks prior to study terminus (32, 33). Thus, we predicted that at least some of the DNA methylation patterns would be maintained after exposure cessation to provide mechanistic insight into the persistence of TCE's effects.

## Materials and Methods

### Mice and TCE Exposure

This study was conducted at ACRI under an approved Animal Use Protocol by the Animal Care and Use Committee at the University of Arkansas for Medical Sciences. Eight weeks old lupus-prone female MRL+/+ mice were purchased from Jackson Laboratories, Bar Harbor, ME, USA. Randomized mice were paired with age-matched male MRL +/+ mice as described (32, 33). Mice were divided into groups that were given ultrapure unchlorinated drinking water (MilliQ) with vehicle only or 500 μg/ml TCE (10 females per group). Vehicle controls were given water containing only 1% Alkamuls EL-620, the reagent used to solubilize the TCE. The drinking water bottles were changed 3 times/week to offset degradation of TCE. The level of direct TCE exposure in offspring (μg/kg/d) from weaning to PND 154 was based totally on ingestion (e.g., average body weight over time and average consumption of water). Female offspring were weighed weekly and water consumption was monitored. TCE exposure (μg/kg/day) was based on the average amount of TCE-containing water consumed per cage divided by the average mouse weight per cage and a previously calculated 20% degradation of TCE in water bottles. On average, the mice that were directly exposed to TCE (continuous exposure) were exposed on average to <100 mg/kg/day, which approximates the current US Occupational Safety and Health Administration (OSHA) Permissible Exposure Limit at 100 ppm or ~76 mg/kg/day (34).

### Study Design

**Figure 1:**
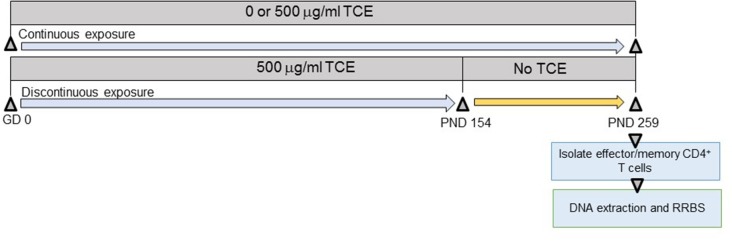
Experimental design. Prior to breeding, female MRL+/+ mice were randomly assigned to treatment groups consisting of (1) vehicle control; (2) 500 μg/ml TCE. Female offspring were exposed continuously during development during gestation, lactation, and directly via the drinking water during early life. At PND 154 TCE was removed from the drinking water from a subset of the offspring. The remaining mice continued vehicle or TCE in the drinking water until PND 259 when all animals were euthanized. CD4^+^ T cells were purified into effector/memory subsets and subjected to DNA extraction and RRBS analysis as described in the methods.

As shown in Figure 1, offspring derived from 8 dams/treatment group were exposed both directly and indirectly to TCE beginning at gestational day (GD) 0 to postnatal day (PND) 0 *in utero*, and then from PND 1 to PND 21 via lactation. Female pups were weaned at PND 21 and further exposed to TCE directly in their drinking water until PND 154 (~22 weeks of age) after which they were administered ultrapure drinking water without TCE until PND 259 (discontinuous group) as described (32). A subset of pups remained on TCE-containing water for an additional 15 weeks until study terminus [PND 259 (continuous group)]. A third group of mice were exposed to vehicle only for the duration of the experiment. PND 259 (~37 weeks of age) was chosen as the endpoint based on previous studies where mice at approximately this age after chronic adult only exposure developed autoimmune pathology and global DNA methylation alterations in CD4^+^ T cells (27). The period for stopping the exposure at PND 154 (~22 weeks of age) was selected based on a previous study that documented a persistence in TCE-induced changes in ~27 week old mice after ~17 weeks of exposure cessation (35).

### Isolation of Effector/Memory CD4^+^ T Cells

Splenic effector memory (CD62L^lo^) CD4^+^ T cells were collected from euthanized mice at study terminus using Dynabeads FlowComp Mouse CD4 kit (Invitrogen) as described (28). The CD4^+^ T cells were then further separated into naïve or effector/memory CD4^+^ T cell populations using Dynabeads M-280 Streptavidin (Invitrogen) conjugated with biotinylated anti-CD62L antibody (eBiosciences, 13-0621-85). The resulting CD62L^lo^ CD4^+^ T cells were stimulated with immobilized anti-CD3 antibody and anti-CD28 antibody overnight and the activated cells were frozen for examination of DNA methylation. To ensure sufficient cells for use in all the assays, each sample of CD4^+^ T cells used in the study originated in an equal number of pooled spleen cells from 2 to 3 female mice per litter resulting in 3–4 samples each from individual litters per each treatment group (3 control samples, 3 discontinuous samples, and 4 continuous samples).

### DNA Methylation Analysis by Reduced Representation Bisulfite Sequencing (RRBS)

DNA from the CD4^+^ T cells was isolated as described (28) using PureLink Genomic DNA Mini Kit (Thermo Fisher Scientific). Purity was examined on the NanoDrop 2000c for an A260/A280 range of 1.8–2.0. DNA quality confirmed using standard gel electrophoresis. The DNA was then restriction digested, end-repaired, purified, and ligated with barcode adapters. The RRBS libraries were generated, bisulfite converted, PCR enriched, size selected, purified, and sequenced (2 × 100 paired end) using Illumina HiSeq sequencer.

The Illumina fastq files were first checked for quality using Babraham Bioinformatics *FastQC* (version 0.11.7). Sequencing adaptors, low quality reads (Q <20), and the ends were trimmed using *Trim Galore* (https://www.bioinformatics.babraham.ac.uk/projects/trim_galore/) and the quality was confirmed using *FastQC*. Bisulfite treated reads were aligned to the m10 reference genome and cytosine methylation sites were called using *Bismark* (https://github.com/FelixKrueger/Bismark). The alignment was performed using bismark with bowtie2 and methylation calls were performed using bismark methylation extractor. The bismark methylation extractor was run with the parameter—no_overlap to ensure that overlapping reads from the paired reads were not measured twice in the final analysis. The bismark coverage files were then imported into R for further analysis.

### Bioinformatics Analysis and Assessment of Global and Differentially Methylated Regions

Methylation levels were also investigated based on the distance to the nearest transcriptional start site (TSS) and plotted using the lowess function in R. The overall global methylation patterns were averaged over all genes. Negative distances correspond to CpG sites downstream of gene TSS. The coefficients of the glmLRT models generated for each comparison were used to assess changes in methylation patterns relative to control as described (36).

The bismark coverage bed files for each sample were generated from Bismark methylation extractor and imported into R in order to identify differentially methylated regions (DMRs) between the control sample TCE dose (continuous), and between the control sample and the TCE dose that was discontinued at PND154 (discontinuous). The analysis was performed as described using the *edge*R Bioconductor package (36, 37) *edge*R is based on the negative binomial distribution and models the variation between biological replicates through the negative binomial dispersion parameter. As opposed to other methylation sequencing data analysis methods, this workflow keeps the counts for methylated and unmethylated reads as separate observations and does not limit analysis to percent methylation. Linear models are then used to fit the total read count (methylated plus unmethylated or M+U) at each genomic locus and methylated reads are modeled indirectly as an over-dispersed binomial distribution. DMRs are assessed by generalized linear models with likelihood ratio tests using edgeR generalized linear model likelihood ratio test (glmLRT). The *p*-values were corrected using the false discovery rate.

The total counts matrix was created by identifying CpG sites present in at least one sample and extracting the read counts of both methylated and unmethylated Cs at each CpG site within each sample. The data was analyzed for individual CpG sites as well as grouping the sites by CpG islands, promoter regions, and by regulatory elements based on the UCSC oregano track. The data was then filtered by requiring a CpG site to have a total count (M+U) of at least 8 across all the samples before it was included in the analysis. CpG islands, promoters, and regulatory elements were required to have a total count of at least 20, 50, and 30 total counts across all of the samples, respectively. The count matrix was then normalized so that the methylated and unmethylated reads were treated as a single unit, and the library sizes were set to be equal for each pair of libraries (average of the methylated and unmethylated library sizes). Both β-values (M/M+U) and M-values (log2 M/U) were calculated for each sample and compared. shinyCircos was used to generate a circos heatmap for the log2 fold-change values in order to visually represent the changes in methylation of promoters and regulatory elements (38).

### Annotation of CpG Sites and Pathway Analysis

Several genomic regions of interest, including CpG islands, promoters, and regulatory elements were analyzed between the different sample groups by annotating with different UCSC genome browser tracks. Individual CpG islands were annotated with the gene with the nearest TSS. Promoters were analyzed using Bioconductor Open Source Software for Bioinformatics Annotation Package (*TxDb.Mmusculus.UCSC.mm10.knownGene package)*. We defined the promoter of a gene as the region from 2 kb upstream to 1 kb downstream of the transcription start site (TSS) of that gene. We also analyzed regulatory elements including transcription factor binding sites, RNA binding sites, regulatory variants, and other regulatory elements utilizing the Open Regulatory Annotation database [(ORegAnno) (39)]. Once the counts matrix was annotated by each track and region of interest, the GLM-likelihood ratio test was applied to identify significantly differentially methylated regions in the continuous and discontinuous TCE dose samples. DMRs were considered significant by a FDR adjusted *p*-value ≤ 0.05 and fold change > 2. GO terms overrepresentation analysis for molecular function and biological process of the significant promoter regions used the groupGO function of the clusterProfiler R package The KEGG pathway analysis was performed using clusterProfiler's gseKEGG function with default parameters (40).

## Results

### Assessment of Global Methylation Patterns

**Figure 2:**
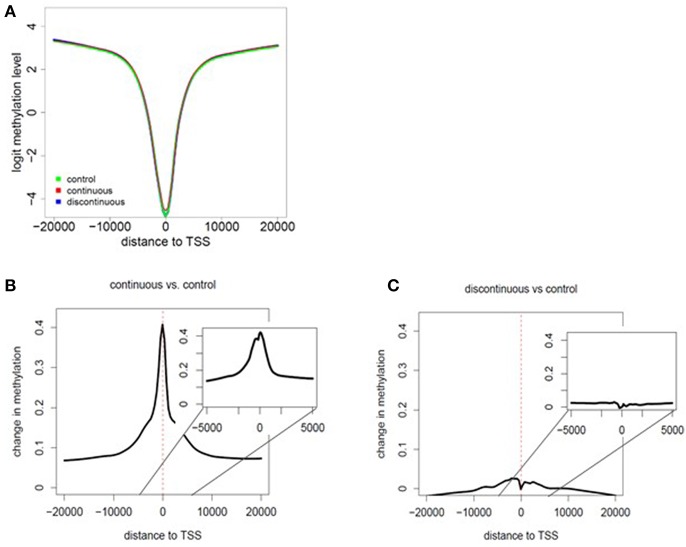
Assessment of global methylation patterns. **(A)** Methylation levels averaged over all genes were investigated based on the distance to the nearest TSS (± 20,000 bp of the TSS for each group). The data in the histogram overlay depicts the methylation level from each exposure group. **(B,C)** Global changes in methylation with continuous exposure **(B)** or discontinuous exposure **(C)** relative to controls are presented in histogram plots. The inset shows the average change in methylation ± 5,000 bp around the TSS. Negative distances correspond to CpG sites downstream of gene TSS. The coefficients of the glmLRT models generated for each comparison were used to assess change in methylation patterns relative to control.

As shown in Figure 1 and described in the methods section, we performed RRBS analysis of purified effector/memory (CD44^hi^/CD62L^lo^) CD4^+^ T cells from female MRL+/+ mice exposed to TCE as described previously (32, 33). All mice were euthanized at PND 259. The RRBS DNA methylation data was quality checked and filtered prior to differential analysis. Global methylation patterns in effector/memory CD4^+^ T cells were analyzed using fry gene set analysis to identify differences in the methylation patterns at the TSS between continuous or discontinuous exposures compared to the control group. Overall, 288,894 CpG sites were assessed in the analysis. The methylated cytosine counts were summed across all genomic regions. Figures 2A–C shows histogram plots of global methylation patterns across all genes ± 20,000 bp of the TSS for each group. The plots showed that regardless of treatment, the basic shape of the methylation distribution did not differ among the groups (Figure 2A). The histogram also revealed that most of the methylated CpG regions were furthest from the TSS, and a majority of the unmethylated CpGs were closest to the TSS regardless of TCE exposure. This pattern was similar when CpG islands, promoters, regulatory elements, and chromosomes were assessed individually (data not shown). Thus, CpG methylation levels in effector/memory CD4^+^ T cells on a global scale were similar among all groups.

Global changes in methylation with continuous exposure (Figure 2B) or discontinuous exposure (Figure 2C) relative to controls are presented in histogram plots. The results revealed that effector/memory CD4^+^ T cells have greater differences in methylation when TCE exposure was continuous. In marked contrast, there was very little change in the levels of methylation relative to controls in CD4^+^ T cells isolated from mice whose TCE was removed from the drinking water ~15 weeks before study terminus. While the greatest changes in methylation occurred in regions closest to the TSS in both groups, this effect was more evident in the continuous exposure group implying increased potential for altered gene expression.

### Assessment of Differentially Methylated Regions in Promoters and CpG Islands

Several genomic regions of interest, including CpG islands, promoters, and ORegAnno regulatory elements were analyzed between the different sample groups by annotating with different UCSC genome browser tracks. Each genomic regions provides unique insight into how DNA methylation alterations regulate gene expression as either individual CpG sites or as a region. For instance, CpG methylation in promoter regions is often associated with silencing of transcription and gene expression. The methylation count data was analyzed using edge R glmLRT as described in the methods section to identify differentially methylated regions (DMRs) associated with each of the UCSC genome browser tracks.

**Figure 3:**
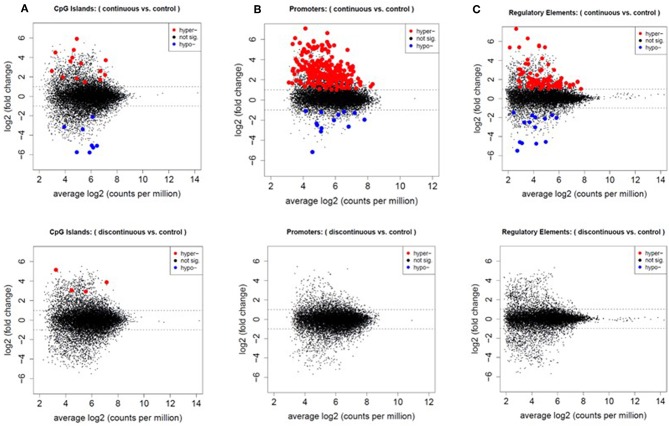
Increased number of differentially methylated regions in the continuous exposure group. CpG sites were grouped by CpG islands **(A)**, promoter regions **(B)**, and regulatory elements **(C)** as described in the methods. Differentially methylated regions were analyzed using edgeR glmLRT. Graphs of the MD-plots represent the fold change (log2) between either continuous vs. control or discontinuous vs. control. DMRs were considered significant with an FDR-adjusted *p* ≤ 0.05 and fold change > 2.

First, we assessed the methylation of CpG islands in each exposure group. CpG sites overlapped with 15,788 of the 16,023 CpG islands in the mouse genome. After filtering for low counts, 13,084 CpG islands (82.9%) remained for DMR analysis. Figure 3, represents mean difference (MD) plots of the DMRs in CpG islands, promoter regions, and regulatory elements. Regions found to be significantly hypermethylated or hypomethylated in the exposure group compared to controls are depicted in red and blue, respectively. As shown in Figure 3A and in Table 1, a majority of the DMRs in both the continuous and discontinuous groups in CpG islands were hypermethylated (69%) relative to controls. Fourteen CpG island regions were significantly hypermethylated, and nine CpG islands were hypomethylated with continuous TCE exposure. In the discontinuous group, only four CpG island regions were significantly hypermethylated relative to controls. As shown in Table 1, among the four hypermethylated CpG islands in the discontinuous group, two regions were also hypermethylated in the continuous exposure group; namely, CpG 69 on chromosome 8 (3.72- vs. 3.88-fold; continuous vs. discontinuous, respectively), and CpG 24 on chromosome 9 (4.52- vs. 5.12-fold; continuous vs. discontinuous, respectively). Interestingly, both of these island regions have been previously linked to PcG-mediated H3K27 tri-methylation, an epigenetic repressive mark (41).

**Table 1:** DMRs in CpG islands.

**Chr**	**ID**	**Start**	**End**	**#CpG**	**%CpG**	**#Gc**	**%Gc**	**Fold change (log2)**	**FDR**
**Continuous**									
Chr19	35	61266505	61266971	466	15	242	51.9	5.932811	0.012014
Chr15	61	76904006	76904671	665	18.3	404	60.8	4.784853	1.69E-05
Chr9	24	78427458	78427765	307	15.6	185	60.3	4.521959	0.005407
Chr9	18	58247028	58247256	228	15.8	138	60.5	4.004769	0.029688
Chr8	69	19784572	19785241	669	20.6	454	67.9	3.72208	1.69E-05
Chrx	128	6172298	6173448	1150	22.3	716	62.3	3.621685	0.014121
Chr9	83	1.09E+08	1.09E+08	929	17.9	570	61.4	3.406221	0.020168
Chr6	45	1.13E+08	1.13E+08	422	21.3	285	67.5	2.611359	0.014121
Chr9	233	95405215	95407761	2546	18.3	1698	66.7	2.602433	0.017659
Chr19	62	8888448	8889270	822	15.1	462	56.2	2.215014	0.029688
Chr2	23	75981547	75981815	268	17.2	162	60.4	1.970652	0.051439
Chr7	93	97325217	97326011	794	23.4	564	71	1.842948	0.020168
Chr5	43	1.24E+08	1.24E+08	365	23.6	252	69	1.735225	0.005407
Chrx	52	36111947	36112448	501	20.8	363	72.5	1.472045	0.012014
Chr6	53	1.41E+08	1.41E+08	367	28.9	258	70.3	−5.7822	0.005281
Chr2	54	34870778	34871358	580	18.6	370	63.8	−5.73915	0.004199
Chr1	81	1.61E+08	1.61E+08	887	18.3	564	63.6	−5.27595	0.029688
Chr15	94	76080388	76081110	722	26	512	70.9	−5.0925	0.048491
Chr3	37	27182877	27183286	409	18.1	262	64.1	−5.06582	0.031513
Chr18	53	74267931	74268512	581	18.2	383	65.9	−3.37984	0.038562
Chr5	17	1.35E+08	1.35E+08	226	15	140	61.9	−3.15186	0.026914
Chr10	70	22273321	22274267	946	14.8	582	61.5	−2.11188	0.051439
**Discontinuous**									
Chr9	24	78427445	78427765	307	15.6	185	60.3	5.159825	0.000668
Chr8	69	19784572	19785241	669	20.6	454	67.9	3.886291	0.000102
Chr11	32	83444820	83445050	230	27.8	157	68.3	3.056442	0.020591
Chr7	30	5125832	5126107	275	21.8	188	68.4	2.931381	0.028506

*Pink represents hypermethylation and blue represents hypomethylation*.

In the discontinuous group, two additional CpG islands were hypermethylated relative to controls that were not altered in the continuous group (Table 1). One region, CpG 32 on Chr11 encodes matrix metalloproteinase 28 (epilysin) or MMP-28. Although not thoroughly studied in T cells, MMP-28 has been shown to be a key regulatory of inflammation and macrophage differentiation (42). The other distinct island region, CpG 30 on Chr7, is a binding site for the transcription factor, Bhlhe40, known to control cytokine production by T cells and has been identified as a critical regulator of autoreactive T cell pathology (43).

In the promoter regions 2,080,592 CpG sites overlapped with 23,711 of the 24,044 promoter regions in the UCSC knownGenes track. After quality filtering, 13,322 promoter regions were analyzed. In the continuous exposure group, 252 CpGs were significantly differentially methylated compared to controls (Figure 3B). Out of these CpGs, 239 (95%) were hypermethylated and only 13 (5%) were hypomethylated relative to control. In contrast, none of the promoter regions were significantly altered relative to controls in the discontinuous group.

### Differentially Methylated Regions Regulatory Elements Were Enriched for Polycomb Protein Binding Sites

**Figure 4:**
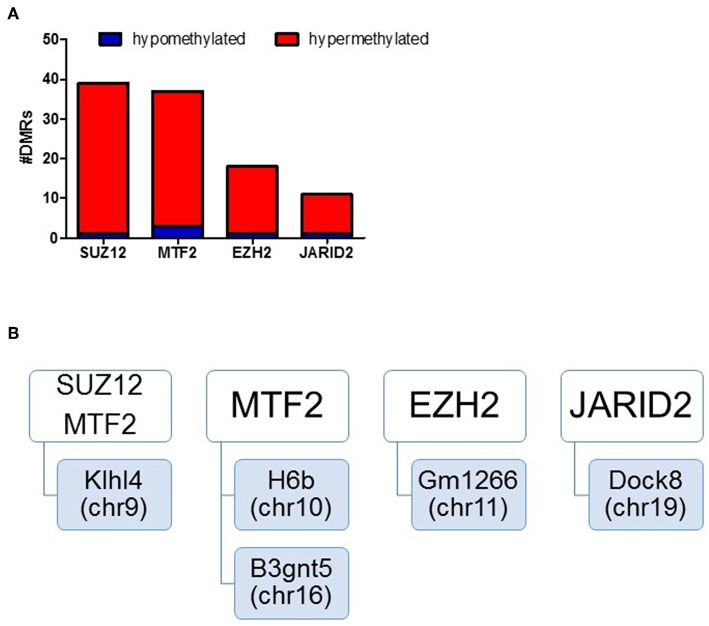
DMRs of PcG binding sites in continuous exposure group are predominantly hypermethylated. RRBS analysis of effector memory CD4^+^ T cells from control and continuous TCE exposure. **(A)** Regulatory elements included in the ORegAnno database were assessed. The number of hypermethylated vs. hypomethylated DMRs for each PcG protein binding region are shown. **(B)** Represents a summary of the few hypomethylated regions according to gene and chromosome location.

**Figure 5:**
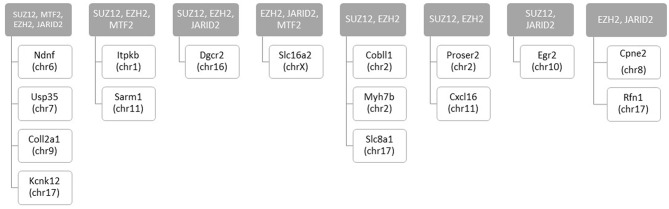
Number of hypermethylated DMRs that are linked to more than one PcG binding sites with continuous exposure. RRBS analysis of effector memory CD4^+^ T cells from control and continuous TCE exposure revealed 129 statistically significant DMRs from regulatory elements based on ORegAnno database. The hypermethylated DMRs associated with PcG group binding sites (*n* = 99) were linked to 16 unique genes associated with more than 1 PcG protein binding site.

We assessed regulatory elements included in the ORegAnno database. After filtering regions with low counts, 13,500 of the 415,390 elements were included in DMR analysis. As shown in Figure 3C, removal of the TCE from the drinking water did not significantly alter the methylation pattern of the CD4^+^ T cells compared to control. However, continuous TCE treatment resulted in 113 hypermethylated and 16 hypomethylated elements. These binding sites included 12 unique transcription factors and regulatory elements. Interestingly, 80% of these DMRs included binding sites for PcG proteins associated with PRC2, namely; SUZ12, EZH2, JARID2, and MTF2. A majority of these PcG enriched DMRs were hypermethylated (*n* = 99) and only 6 were hypomethylated (*n* = 6). Figure 4A shows the number of hypermethylated vs. hypomethylated DMRs for each PcG protein binding region. Figure 4B breaks down the six hypomethylated regions according to gene and chromosome location. The *klhl4* gene on chromosome nine was linked to two PcG protein binding sites, SUZ12 and MTF2. Based on this finding, we analyzed the other 99 hypermethylated DMRs associated with PcG protein binding to see if any of the genes were linked to a shared region. Figure 5 shows that among these regions, 16 unique genes linked to 44 DMRs were associated with more than one PcG protein binding site.

The other statistically significant DMRs included transcription factors representing 12 sites including; EBF1 (Early B Cell Factor 1), GATA1 (GATA Binding Protein 1), FOXA2 (Forkhead Box A2), STAT1 (Signal Transducer and Activator of Transcription), ATOH1 (Atonal BHLH Transcription Factor 1), ESR1 (Estrogen Receptor 1), KLF1 (Kruppel Like Factor 1), and EGR2 (Early Growth Response 2) (Table 2). Although there were fewer significant DMRs in transcription factor binding sites compared to PcG binding regions; a greater percentage of the DMRs in the transcription factor binding sites were hypomethylated; 14 of the transcription factor binding sites were hypermethylated and 10 regions were hypomethylated. Depending on the chromosomal location, five transcription binding sites were both hyper- and hypomethylated (GATA1, FOXA2, EBF1, and STAT1). ATOH1 was only hypermethylated (on chr7 and chr5), and three transcription factors (KLF1, ESR1, and EGR2) were exclusively hypomethylated.

**Table 2:** Transcription factors associated with DMRs from regulatory regions in the continuous exposure group.

**Oreg ID**	**TF binding site**	**Gene**	**Chr**	**Start site**	**Fold change (log2)**	**FDR**
OREG1667709	GATA1	Got1	chr19	43524408	5.400330	0.0378661
OREG1667084	GATA1	Ntpcr	chr8	125734385	5.3773777	0.0531756
OREG0071054	FOXA2		chr2	155050089	4.223175	0.001133
OREG1562375	EBF1	Ryr1	chr7	29040109	3.087317	0.0300861
OREG1666877	GATA1	Cyb5b	chr8	107150438	3.0108383	0.0451529
OREG1201127	ATOH1	Gm12764	chr7	34390804	2.130084	0.00403
OREG1559800	EBF1	Btd	chr14	31660270	1.797766	0.028670
OREG1564020	EBF1	Vps33a	chr5	123572681	1.742096	0.000369
OREG0048798	FOXA2	Gfil	chr5	107687900	1.460722	0.0328458
OREG1864590	STAT1	Gfi1	chr5	107688001	1.423059	0.0300861
OREG1199817	ATOH1	Gfi1	chr5	107688015	1.422497	0.024385
OREG0048387	FOXA2		chr4	150889206	1.3308524	0.0406727
OREG1665142	GATA1	Pde4dip	chr3	97767156	1.271897	0.021289
OREG1562020	EBF1	Cnksr3	chr10	7158580	1.2246290	0.0514763
OREG1760351	KLF1	Fig4	chr10	41204944	−5.4811978	0.0298196
OREG1668792	GATA1	Fig4	chr10	41204856	−5.4811978	0.0298196
OREG1563191	EBF1	Flcn	chr11	59809474	−4.7241132	0.0201386
OREG0042314	FOXA2	Ddx42	chr11	106216425	−4.6945932	0.0153887
OREG1863898	STAT1	Ddx42	chr11	106216435	−4.5741675	0.0221824
OREG0035646	ESR1		chr11	116199352	−4.54767328	0.0270150
OREG0598482	EGR2		chr2	144599905	−3.01723545	0.0286700
OREG1869968	STAT1	Eif4ebp3	chr18	36663812	−2.50431736	0.0300861
OREG0037174	ESR1	Eif4ebp3	chr18	36663725	−2.50431736	0.0300861
OREG1561198	EBF1	Slc1a1	chr12	80998351	−1.46607522	0.0220945

*Pink represents hypermethylation and blue represents hypomethylation*.

**Figure 6:**
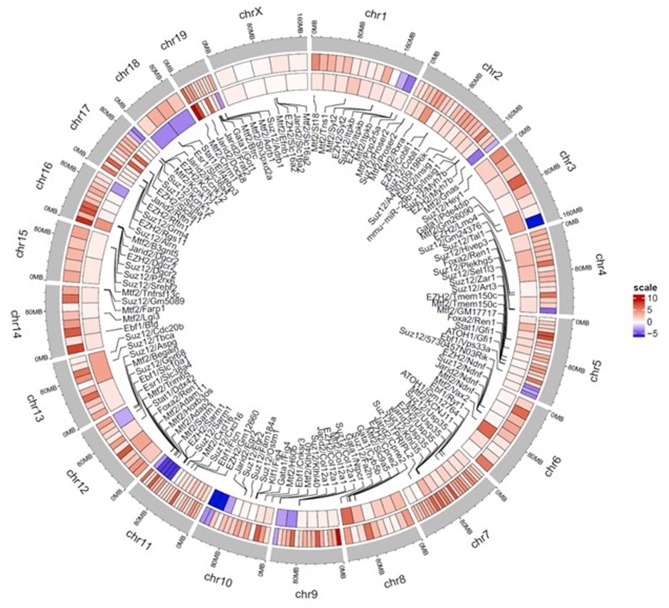
Circos plot heatmap showing gene promoter and regulatory element regions that were significantly hyper or hypo-methylated with continuous TCE exposure. The heatmap indicates the average Log 2 fold change values for the statistically significant DMRs of promoters (outer ring) and regulatory elements (innermost ring). The outer most ring (gray) shows the chromosome and location. The lines point to associated regulatory element and chromosome location. Red indicates hypermethylation and blue indicates hypomethylation relative to controls.

A circos heatmap of the log2 fold change values in the continuous treatment compared to controls was generated to summarize the data. Figure 6 depicts regional location and chromosomal location of hyper- and hypo-methylated DMRs in both promoters (outer ring) and regulatory elements (inner ring). Overall, the DMRs were predominantly hypermethylated (red) as opposed to only a few blue regions indicating hypomethylation. Thus, based on the overall hypermethylation of genes in regulatory elements and promoters, continuous TCE treatment may favor overall suppression of transcription compared to controls. The complete list of genes and chromosomal location for all DMRs linked to promoters and regulatory regions are found in Supplementary Table 1.

### Pathways and Genes Associated With DMRs

**Figure 7:**
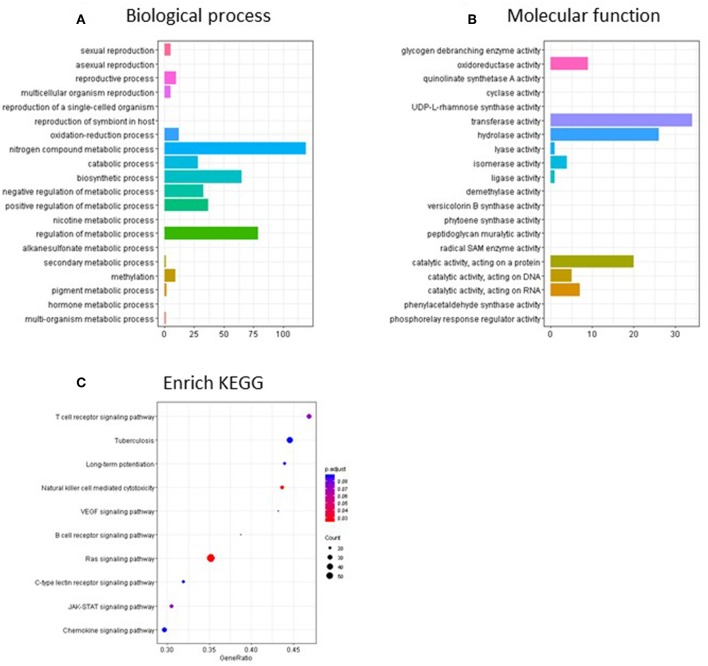
Go terms and KEGG enriched pathways in the promoter regions of the continuous TCE group compared to control. GO terms overrepresentation analysis for **(A)** biological process and **(B)** molecular function of the significant promoter regions used the groupGO function of the clusterProfiler R package as described in the methods. **(C)** The KEGG pathway analysis was performed using clusterProfiler's gseKEGG function with default parameters.

Pathway analysis of the CpG sites differentially methylated by continuous TCE exposure in promoter regions revealed that, in terms of biological processes (Figure 7A), TCE primarily altered the methylation of genes associated with regulation of cellular metabolism, catabolism, and biosynthesis. Focusing in on the molecular function category, TCE effects were associated with transferase/hydrolase and cataloytic activity that were in turn, enriched for genes associated with various functions including cellular signaling pathways (Figures 7B,C). Taken together, TCE altered DNA methylation in a manner that seemed primed to impact downstream gene expression.

## Discussion

Sensitivity to immunotoxicants has been shown to be greater in animal models if exposure occurred during development compared with adulthood (44). In humans, it is not uncommon for IMIDs to manifest during childhood or adolescence suggesting developmental origins (45). These effects are believed to be due, in part, to epigenetic alterations including aberrant DNA methylation (46–50). DNA methylation is crucial for normal functioning of T cells during development. For example, CpG demethylation is important during T cell maturation in the thymus related to TCR function (51). In early life, the phenotype of CD4^+^ T cell subsets that have differentiated in response to antigen are normally controlled by carefully maintained levels of DNA methylation in pertinent regulatory genes (52, 53). Throughout the lifespan, regulation of Th subset differentiation and expansion of effector/memory CD4^+^ T cells is DNA methylation dependent (54). Thus, any event that perturbs the methylome may have important consequences for CD4^+^ T cell function and disease. Aside from TCE, a role for environmental chemical exposures during development including mercury, dioxin, and bisphenol-A have been shown to alter DNA methylation and promote autoimmunity (55).

In the current study, it was predicted that a continuous exposure to TCE administered during development and early life would have a greater impact on the DNA methylation patterns of activated effector/memory CD4^+^ T cells compared to adult-only exposure (28). Overall, TCE-mediated effects reported in this study and our previous study were strikingly similar. Apparently, TCE exposure, regardless of whether it began during development or adulthood, was associated with enrichment of regions of the genome linked to PcG group binding in effector/memory CD4^+^ T cells. Another consistent finding between the two studies was that CpG methylation in activated effector/memory CD4^+^ T cells favored hypermethylation rather than hypomethylation. Thus, the results of two independent studies involving chronic low-level TCE exposure were relatively consistent.

In addition to global changes we focused on specific genomic regions of interest, including CpG islands, promoters, and ORegAnno regulatory elements by annotating with different UCSC genome browser tracks to understand how alterations in DNA methylation may regulate gene expression on an individual CpG level or as a region. When these regions were assessed, a total of 403 significant DMRs were identified in the continuous exposure group relative to controls after quality filtering; almost twice as many significant DMRs identified than in our previous investigation (28).

The complex interplay between DNA methylation and PRC2 binding and its functional consequences in T cells are only beginning to be studied. Among the PRC2 components that regulate PcG function, EZH2 is perhaps the most widely studied in T cells where it is highly expressed (56). Loss of EZH2, which enables functional inactivation of PRC2 and reduction of H3K27me3 levels, has been shown to promote autoimmune pathology commensurate with a reduction in T reg numbers (57) Three independent groups reported decreased IFN-γ production from PRC2/EZH2 deficient T cells cultured under Th1 polarizing conditions (31, 58, 59). One other report showed that EZH2 increased the stability of T-bet, an important Th1 transcription factor (60). Together, these reports underscore the importance of PcG proteins in Th1 differentiation, and suggest that a TCE-mediated alteration in PRC2 binding and downstream upregulation of proinflammatory Th1 cytokines could play a role in the ability of TCE to promote autoimmunity.

Additional work is needed to understand how TCE exposure modulates EZH2 and other associated PcG proteins in the PRC2 complex (e.g., SUZ12, MTF2) whose binding to DNA due to TCE-mediated DNA hypermethylation may be compromised. Indeed stability of PRC2 is dependent on several different components. PRC2 is stably recruited by MTF2 with JARID2, and are important in establishing repressive domains across the genome. In *mtf2* knockout cells, EZH2 catalytic subunit is abrogated, resulting in greatly reduced H3K27me3 deposition (61). Recently, a live imaging study underscored the importance of SUZ12 interaction with several accessory proteins for PRC2 chromatin binding *in vivo* (62). Thus, it is apparent that these PcG proteins are all indispensable functionally for PRC2. Our finding that many of the DMRs associated with TCE exposure bound more than one PcG binding site underscores a need to further study the complex interplay between TCE-induced DNA methylation events and PcG proteins.

In addition to PcG protein binding regions, in the remaining 20% of DMRs, 12 transcription factors were identified as being differentially methylated in the continuous exposure group. All of these transcription factor binding sites have been shown to potentially alter T cells and/or affect subset differentiation, and some have been implicated in autoimmunity. For example, FOXA2 is has been shown to regulate T cell differentiation in the thymus to promote positive selection of CD4^+^ T cells while downregulating Tregs (63). FOXA2 modulated the production of Th2 cytokines in mouse atopic dermatitis model via its action on T regs (64). In the current study, FOXA2 was both hypermethylated and hypomethylated in different regions. In fact, we showed that, unlike the PcG sites, there were almost as many hypomethylated regions as hypermethylated regions including 3 transcription factor binding sites that were exclusively hypomethylated (e.g., KLF1, ESR1, and EGR2) with continuous exposure. Although the significance of this finding is not clear, all 3 of these transcription factors have been shown to impact some aspect of T cell differentiation and autoimmunity (65–67).

The study also included an evaluation of activated effector/memory CD4^+^ T cells isolated from mice after TCE was removed for a period of 15 weeks prior to study terminus. Persistence of functional effects in adult mice after developmental exposure followed by removal of TCE from the drinking water has been reported in previous studies (35, 68) including the mice that were used for a source of effector/memory CD4^+^ T cell DNA for RRBS in the current study (32, 33). These results indicated a unique programming effect of TCE after discontinuation of the dose. Because epigenetic changes are often associated with maintenance of phenotypic effects, we expected to find DNA methylation events would be maintained and perhaps unique methylation profiles linked to these sustained phenotypes would be revealed. However, based on the global methylation pattern and DMR results, very little significant changes in methylation were found in the discontinuous group. It is not clear why the phenotypical effects observed in the same animals did not translate to DNA methylation effects. It is plausible that organ pathology occurred well before the TCE was removed, and events may have been irreversible based on some other mechanism including direct toxicant-induced direct damage or by disrupting repair or anti-oxidant systems designed to promote regeneration and recovery.

Despite the seemingly modest results in the discontinuous group, one interesting finding was the 2 shared hypermethylated DMRs in CpG islands in both the continuous and discontinuous group. The significance of this finding is not clear, but these shared CpG island regions have been associated with histone H3K27 tri-methylation (41). Mammalian PRC2 binding sites in CpG islands have primarily been described in embryonic stem cells where these island regions are generally unmethylated but remain transcriptionally active via H3K27, and have been identified as an important mechanism for expression of tumor suppressor genes (69). In contrast, PRC2 binding does not appear to be restricted to CpG islands in somatic cell types where there is a complex interplay between PRC2 binding and DNA methylation that is not well understood (70). Promoter regions with methylated H3K27 are more likely to gain DNA methylation by PRC2 recruitment of DNA methyltransferases during differentiation (71–73). Taken together, our result would suggest that certain PcG protein-related functions may be impaired due to increased methylation of these regions with continuous exposure. It is possible that at least some of these PcG-mediated functions could be maintained over time after removal of TCE from the drinking water. A closer look at these island regions may indicate a potential mechanism involved in the maintenance of immunotoxicity and autoimmune disease progression in our model.

Limitations of the study included the use of *ex vivo* stimulated CD4s without additional comparisons including assessment of effector/memory CD4 cells that were not stimulated with anti-CD3/CD28. Such an assessment would have provided useful baseline responses. However, this limitation does not affect the significance of the findings of the current study that was designed to directly compare TCE's effects on genome-wide DNA methylation patterns in our published study using only activated effector/memory CD4s from mice that were exposed during development vs. adulthood (28).

Although we expected to observe even more robust changes following a developmental exposure compared to adult only exposure, this study was conducted in already differentiated, activated effector memory CD4^+^ T cells. It is known that epigenetic modifications are more likely to accompany CD4^+^ T cell differentiation, and these dynamic events are difficult to recapitulate *in vivo*. Thus a different approach that includes examining the time-dependent events that accompany CD4^+^ T cell differentiation is necessary to distinguish whether enrichment of PcG proteins in the current study are associated with alterations and corresponding gene in actively differentiating cells. Our results highlight the possibility that similarities observed between continuous developmental vs. adult-only exposure represent marks of a terminally differentiated cell rather than some alteration that is unique to the timing of the exposure. Despite the limitations, the current study demonstrated that TCE regulated PcG binding sites in effector/memory CD4^+^ T cells when exposure occurred continuously throughout development and early life. Future research will continue to explore how these DNA methylation alterations in PcG proteins may promote TCE-induced immunotoxicity with implications for autoimmune disease mechanisms in humans.

## Ethics Statement

This study was carried out in accordance with the recommendations of Animal Welfare Act and PHS Policy on Humane Care and Use of Laboratory Animals. The protocol was approved by the University of Arkansas for Medical Sciences Institutional Animal Care and Use Committee.

## Author Contributions

SBy contributed to the manuscript by overseeing all aspects of data analysis and assisted in writing the manuscript. CW conducted data analysis, assisted in writing of the manuscript, and generated many of the figures presented in the manuscript. JP assisted with organization and analysis of the data. KV conducted animal exposures and prepared the T cells for DNA extraction and library preparation. KG implemented the experimental design and conducted initial data analysis. SBl contributed to the manuscript by implementing the experimental design, interpreting the results, and writing the manuscript.

### Conflict of Interest Statement

The authors declare that the research was conducted in the absence of any commercial or financial relationships that could be construed as a potential conflict of interest.
